# The Combination of *Trichoderma harzianum* and Chemical Fertilization Leads to the Deregulation of Phytohormone Networking, Preventing the Adaptive Responses of Tomato Plants to Salt Stress

**DOI:** 10.3389/fpls.2017.00294

**Published:** 2017-03-02

**Authors:** M. B. Rubio, Rosa Hermosa, Rubén Vicente, Fabio A. Gómez-Acosta, Rosa Morcuende, Enrique Monte, Wagner Bettiol

**Affiliations:** ^1^Spanish-Portuguese Institute for Agricultural Research (CIALE), Department of Microbiology and Genetics, University of SalamancaSalamanca, Spain; ^2^Abiotic Stress Department, Institute of Natural Resources and Agrobiology of Salamanca – Consejo Superior de Investigaciones CientíficasSalamanca, Spain; ^3^Embrapa EnvironmentJaguariúna, Brazil

**Keywords:** abiotic stress, saline, growth promotion, nitrogen fertilizer, phytohormone regulation

## Abstract

Plants have evolved effective mechanisms to avoid or reduce the potential damage caused by abiotic stresses. In addition to biocontrol abilities, *Trichoderma* genus fungi promote growth and alleviate the adverse effects caused by saline stress in plants. Morphological, physiological, and molecular changes were analyzed in salt-stressed tomato plants grown under greenhouse conditions in order to investigate the effects of chemical and biological fertilizations. The application of *Trichoderma harzianum* T34 to tomato seeds had very positive effects on plant growth, independently of chemical fertilization. The application of salt stress significantly changed the parameters related to growth and gas-exchange rates in tomato plants subject to chemical fertilization. However, the gas-exchange parameters were not affected in unfertilized plants under the same moderate saline stress. The combined application of T34 and salt significantly reduced the fresh and dry weights of NPK-fertilized plants, while the opposite effects were detected when no chemical fertilization was applied. Decaying symptoms were observed in salt-stressed and chemically fertilized plants previously treated with T34. This damaged phenotype was linked to significantly higher intercellular CO_2_ and slight increases in stomatal conductance and transpiration, and to the deregulation of phytohormone networking in terms of significantly lower expression levels of the salt overlay sensitivity 1 (*SOS1*) gene, and the genes involved in signaling abscisic acid-, ethylene-, and salicylic acid-dependent pathways and ROS production, in comparison with those observed in salt-challenged NPK-fertilized plants.

## Introduction

Nitrogen is an essential nutrient for plants, and its shortage is a major factor restricting the growth, development and productivity of crops, as this element is often only present in small quantities locally or in a form they cannot use (Kraiser et al., 2011). Over the past 40 years, the amount of synthetic nitrogen applied to crops for increasing their yields has risen from 12 to 104 Tg/year (Mulvaney et al., 2009), but only ca. 30–50% is taken up by the growing plant, with the remainder being lost, which has a very negative impact on the environment (Rockström et al., 2009).

Crop yields are adversely affected by abiotic stresses such as extreme temperatures, drought or high salinity. Soil salinization is a global problem because there are currently more than 34 million hectares (Mha) of irrigated land that have been seriously compromised by the build-up of salts, and 0.25–0.5 Mha are estimated to be lost to production every year (Aquastat, 2016). The increasing salinization rate constantly affects photosynthesis, protein synthesis, and energy and lipid metabolism, and also impacts negatively on chemical and physical soil characteristics, which all combine to reduce plant growth (Allakhverdiev et al., 2000; Parida and Das, 2005; Jesus et al., 2015). However, plants have evolved effective adaptive mechanisms to avoid or reduce the possible damage caused by abiotic stresses. They can perceive adverse environmental conditions and elicit appropriate responses by adjusting their metabolism, growth and development. Phytohormones play key roles in the regulatory circuits in response to adverse environments by mediating specific molecular and physiological changes. Hormones and other signaling compounds are interrelated by synergistic or antagonistic crosstalk (Peleg and Blumwald, 2011; Saxena et al., 2016), and certain phytohormone-regulated mechanisms plants use to adapt to abiotic stress are now being reported (Suzuki et al., 2013; Wang et al., 2014; Saxena et al., 2016). A recent model involving abscisic acid (ABA), reactive oxygen species (ROS) and stomata has been proposed to explain the signaling network leading to systemic acquired acclimation to abiotic stress in plants (Mittler and Blumwald, 2015). Although salicylic acid (SA) has been extensively studied in plant-pathogen interactions because of its involvement in systemic acquired resistance (SAR) (Spoel et al., 2009), the key role it plays in plant responses to abiotic stresses has recently been described (Jayakannan et al., 2015; Zandalinas et al., 2016b).

Under field conditions, plants are usually exposed to a wide range of stimuli, and their responses to stress combinations are not a mere summation of the effects of individual stress factors (Mittler and Blumwald, 2010). Recent studies show that depending on the nitrogen source, plants react in a complex manner to moderate salt stress (Meng et al., 2016) or to a combination of drought and high temperature (Zandalinas et al., 2016b).

Rhizosphere microorganisms contribute to sustainable agriculture, nature conservation, and the mitigation of climate change, ensuring high productivity with low ecological impact (Philippot et al., 2013). Inorganic nitrogen uptake in the rhizosphere of beneficial plant root-bacteria and -mycorrhizal fungi interactions has been well-documented (Courty et al., 2015). In addition, the rhizosphere bacteria and mycorrhizal fungi that interact with plants by alleviating stress constitute a novel strategy against salinity, as well as a tool for understanding how the alleviation of saline stress has beneficial effects on plant growth and productivity (Dimkpa et al., 2009; Evelin et al., 2009; Dodd and Pérez-Alfocea, 2012; Yuan et al., 2016). It is widely recognized that species of *Trichoderma* protect plants against pathogens by competition, mycoparasitism and antibiosis (Lorito et al., 2010; Druzhinina et al., 2011). Moreover, the beneficial effects that *Trichoderma* has on plants, such as promoting growth and inducing defenses against biotic and abiotic damage, are receiving increased attention (Shoresh et al., 2010; Hermosa et al., 2012), and it has been reported that *Trichoderma* spp. are effective in alleviating the adverse effects that saline stress has on plants (Mastouri et al., 2010; Rubio et al., 2014). *Trichoderma*-based products such as biofungicides, biofertilizers or plant strengthening agents are used worldwide in different models of agricultural production (Harman et al., 2010), with *Trichoderma harzianum* being the most frequently cited species as an active matter in a variety of commercial formulations (Morán-Diez et al., 2012). It has also been reported that treating rice, wheat and tomato seeds with *T. harzianum* increases their photosynthetic rate, the weight of the plants, their shoot and root length, and the number of leaves and leaf area (Rawat et al., 2011; Domínguez et al., 2016). Although studies on the role of *Trichoderma* in symbiotic nitrogen assimilation, as a major component of these beneficial responses, are still scarce, in the late 1990s it was reported that *T. harzianum* favored nitrogen uptake in maize field trials (Harman, 2000).

The physiological parameters and expression changes of genes linked to abiotic stresses have been assessed to investigate the responses of chemically fertilized and unfertilized tomato plants to *T. harzianum* T34, saline stress, and the interaction of both factors. Our results indicate that the combination of biological and chemical fertilization increases the sensitivity of tomato plants to salt stress and prevents adaptive responses. However, in the absence of chemical fertilization, *T. harzianum* promotes plant growth, independently of saline stress.

## Materials and Methods

### Microorganisms and Tomato Seeds

*Trichoderma harzianum* CECT 2413 (Spanish Type Culture Collection, Valencia, Spain), isolated by R. Weindling from soil in USA, referred to here as T34 strain, was used throughout this study. The T34 strain was grown on potato-dextrose-agar (PDA, Sigma-Aldrich) in the dark at 28°C, and stored at -80°C in a 20% glycerol solution. Spores from 7-day-old PDA plates were harvested by adding 10 mL of sterile water to the plates and by scraping the culture with a rubber spatula. These suspensions were filtered through a double layer of cheesecloth to separate large mycelial fragments from conidia. Spore concentrations were calculated using a counting chamber.

Tomato seeds (*Solanum lycopersicum* “Marmande”) were surface-sterilized by vigorous sequential shaking in 70% ethanol and 2% sodium hypochlorite solutions for 10 min each, and then washed thoroughly four times in sterile distilled water, and then air-dried on a sterile gauze sheet.

### *In vivo* Assays

The ability of *T. harzianum* T34 to promote the growth of tomato plants and induce salt tolerance under two chemical fertilization conditions was evaluated in *in vivo* assays.

Surface-sterilized tomato seeds were coated with 1 ml of an aqueous suspension containing 1 × 10^8^ spores ml^-1^ of T34, and then air-dried in an open Petri dish overnight under a laminar flow hood. One ml was used to coat 30 seeds. *Trichoderma-*treated tomato seeds were sown in multi-cell growing trays containing a commercial substrate (Projar Professional – Comercial Projar SA – OM = 90%, cinders = 10%, pH = 5.5–6, EC = 1.5–1.8 mS/cm, with 0.8 kg/m^3^ of NPK 14:16:18, plus micronutrients and 3 kg/m^3^ dolomitic calcium). Untreated tomato seeds were used as control. Two-week-old seedlings were transferred to 0.7 l-pots with the same substrate described before (two seedlings per pot), and after a week 1 plant was kept per pot. Pots were kept in plastic trays (10 pots per tray), and 1 | of a solution containing 2 g/l Multi-Feed NPK Fertilizer (Haifa Chemical, Ltd, Israel) [N_Total_ = 14% (N_NH4_ = 6% and N_NO3_ = 8%), P_2_O_5_ = 7%, K_2_O = 28%, MgO = 2%, SO_3_ = 3%, B = 0.01%, Cu = 0.0055%, Fe = 0.05%, Mn = 0.025%, Zn = 0.075%, Mo = 0.0035%] was applied to each tray 10 days after transplanting (24-day-old plants). Eight and 12 days after the application of NPK fertilizer (on 32- and 36-day-old plants, respectively), 2 l/tray of a 300 mM NaCl solution was either applied or not (salt control). The plants were maintained in a greenhouse at 22 ± 4°C, as previously described (Rubio et al., 2012), and watered as needed with 2 l/tray. This experiment consisted of a 2x2 factorial design with *T. harzianum* treatment [-T34 (control)/+T34] and salt application [No (-salt)/Yes (+salt)]. There were 10 replicate plants per condition in a completely randomized design. The experiment lasted 39 days.

A second greenhouse experiment was carried out as described above, except that the NPK fertilizer was not applied. This *in vivo* assay also included 40 plants (10 replicates per condition), and also lasted 39 days.

Gas-exchange measurements were taken 72 h after the first salt application, and leaf tissue was collected for real-time PCR analysis at this time. Stem height, diameter and the fresh weight of plants and the dry weight of aboveground and root tissues were measured when the plants were 39 days old.

### Gas-Exchange Measurements

Gas-exchange measurements [rate of photosynthesis (A_n_), stomata conductance (g_s_) transpiration (E), and intercellular CO_2_ concentration (C_i_)] were conducted with an air flow rate of 293.9 ± 4.8 ml min^-1^, saturating 1,500 μmol m^-2^ s^-1^ irradiance and a 1.19 ± 0.11 kPa vapor pressure deficit, using a 1.7-cm^2^ window leaf chamber connected to a portable infrared gas analyzer – IRGA (CIRAS-2; PP Systems, Hitchin, Herts, UK) with differential operation in an open system. Temperature was kept at 25°C with the analyzer’s Peltier system. Measurements were performed on a clear day between 3 and 8 h after dawn, when peak photosynthesis rates are expected. The measurements were taken on two mature leaves, from an intermediate position on the stem of six replicate plants from each condition considered.

### Real-Time PCR Analysis

Total RNA was extracted using TRIZOL^®^ reagent (Invitrogen Life Technologies, Carlsbad, CA, USA), following the manufacturer’s instructions.

Gene expression was analyzed by quantitative real-time PCR (qPCR). cDNA was synthesized from 2 μg of RNA, which was treated with DNase RQ1 (Promega Biotech Ibérica, Alcobendas, Spain), and then used for reverse transcription with an oligo(dT) primer with the Transcriptor First Strand cDNA Synthesis Kit (Takara, Inc., Tokyo, Japan), following the manufacturer’s protocol. The aerial parts of nine plants (three different sets of three plants each) per condition were collected and pooled for RNA extraction, and subsequent cDNA synthesis. Real-time PCR reactions were performed with a StepOnePlus thermocycler (Applied Biosystems, Foster City, CA, USA) in a total volume of 10 μl, using SYBR FAST KAPA qPCR (Biosystems, Buenos Aires, Argentina), and a final primer concentration of 100 nM each. All the reactions were performed under the following conditions: an initial denaturation step (10 min at 95°C) followed by 40 cycles of denaturation (30 s at 95°C), annealing (1 min at 60°C), and extension (1 min at 72°C). Ct values were calculated using Applied Biosystems software, and Microsoft Excel was used to calculate transcript abundance from Ct (cycle threshold) values normalized to the *actin* gene signal. The slopes and efficiency for each primer pair used were measured for a dilution series of cDNA samples, and calculated using Applied Biosystems software (Supplementary Table S1). Relative expression levels of the genes *AREB2* (ABA-responsive element binding protein 2), *EIN2* (ethylene-insensitive protein 2), *NPR1* (non-expressor of pathogenesis related protein 1), *LERBOH1* (NADPH oxidase 1), *TPX1* (peroxidase), *APX1* (ascorbate peroxidase 1), *ARF1* (auxin responsive factor 1), *SOS1* (salt overlay sensibility 1) and *DREB3* (dehydration-responsive element-binding protein 3) were calculated using the 2^-ΔΔCT^ method (Schmittgen and Livak, 2008).

### Statistical Analyses

Two independent experiments were performed considering the application or not of NPK fertilizer. Data for 10 (growth), six (gas-exchange) and three sets of three plants each (qPCR) were analyzed using Minitab 14.20 statistical software, through an analysis of variance (ANOVA) to test for possible interactions between the main effects, followed by a mean separation using Tukey’s test (*P* < 0.05). To analyze the phytohormone-related gene expression in plants subjected, or not, to salt stress, each data set was submitted to a one-way ANOVA and means compared by Tukey’s test (*P* < 0.05) using Statistica 7 software.

## Results

### Effect of T34 and Salt Application on NPK-Fertilized Tomato Plants

Twenty-four-day-old tomato plants derived from seeds coated with conidia of *T. harzianum* T34, or not (control), were subjected to NPK fertilization, and 8 days later a dose of two liters of 300 mM NaCl was applied or not per tray containing 10 plants. Two days later, unexpected decay symptoms were detected in NPK-fertilized tomato plants derived from T34-treated seeds in response to salt application. Compared to NPK-fertilized control, the gas-exchange parameters recorded in 35-day-old plants (72 h after salt application) at saturating irradiance for the T34 treatment showed that this fungus tends to increase the photosynthetic rate by 9.1%. This increase was accompanied by 24.9, 19.3, and 8.8% reductions in stomatal conductance, transpiration, and intercellular CO_2_ concentration, respectively (**Table 1**). Independently of the salt application, T34 did not modify any of the gas-exchange parameters measured. However, the saline treatment significantly reduced photosynthesis, stomatal conductance and transpiration, regardless of whether the tomato seeds were treated with T34, while a salt and T34 combination led to a significant increase in intercellular CO_2_ concentration, and even a non-significant increase in stomatal conductance (42%) and transpiration (39%). These parameters were compatible with the decaying phenotype observed for the T34+salt condition.

**Table 1:** Gas-exchange measurements in NPK-fertilized tomato plants.

Treatment	A_n_	g_s_	E	C_i_
Control	6.47	146.9 c	1.61	285 ab
T34	7.06	110.3 b	1.30	260 a
Salt	2.14	25.5 a	0.31	249 a
T34+Salt	2.08	36.1 a	0.43	314 b
*P*_T34 × Salt_	ns	^∗^	ns	^∗∗^
*P*_T34_	ns	ns	ns	ns
*P*_Salt_	^∗∗∗^	^∗∗∗^	^∗∗∗^	ns


*Rate of photosynthesis (A_*n*_), stomatal conductance (g_*s*_), transpiration (E) and intercellular CO_*2*_ concentration (C_*i*_) were recorded in plants derived or not from *Trichoderma harzianum* T34-treated seeds, and with or without salt stress (2 l of a 300 mM NaCl solution per 10 plants). NPK fertilizer was applied to 24-day-old plants, and salt was applied to 32-day-old plants. Measurements were taken 72 h after salt application. Each value is the mean of two measurements taken for six replicates (*n* = 6), and are expressed as μmol m^-*2*^ s^-*1*^, A_*n*_; mmol m^-*2*^ s^-*1*^, g_*s*_; mmol m^-*2*^ s^-*1*^, E; and μmol mol^-*1*^, Ci. Significant effects were determined by a two-way ANOVA (^∗^*P* < 0.05, ^∗∗^*P* < 0.01, ^∗∗∗^*P* < 0.001) for T34, salt and the T34 × salt interaction, ns, no statistical differences. Different letters denote statistical significance for the T34 × salt interaction.*

**FIGURE 1:**
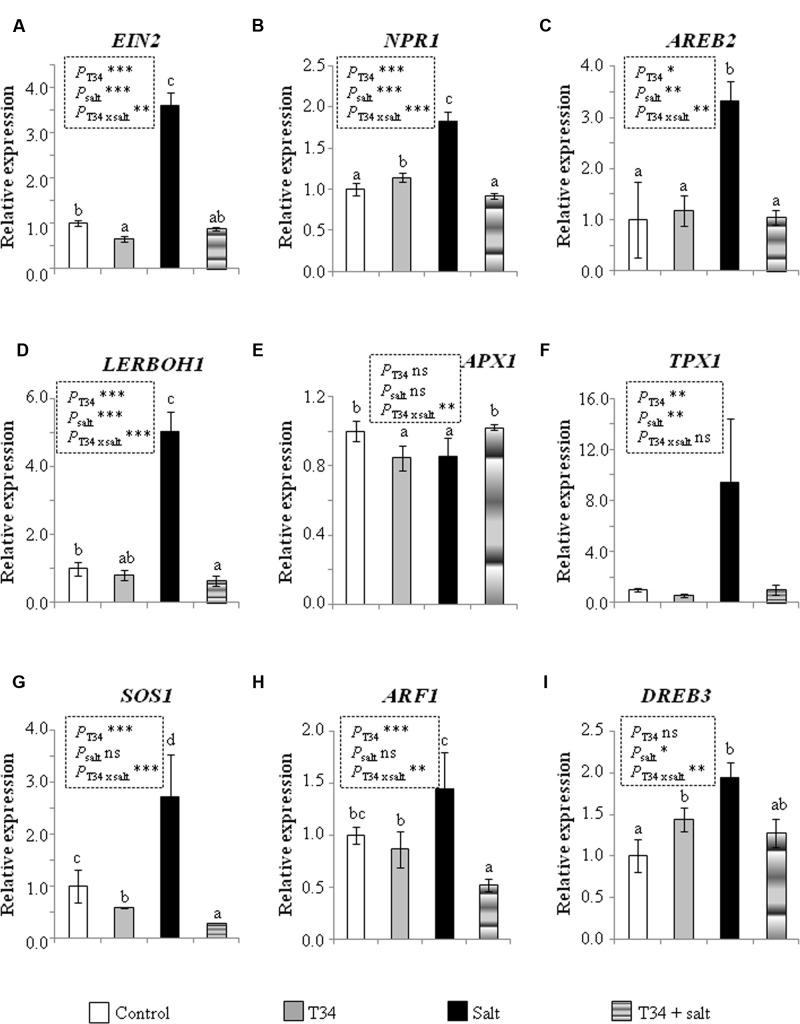
**Relative expression of the genes *EIN*2** **(A)**, *NPR1* **(B)**, *AREB2* **(C)**, *LERBOH1* **(D)**, *APX1* **(E)**, *TPX1* **(F)**, *SOS1* **(G)**, *ARF1* **(H)**, and *DREB3* **(I)** in leaves from 35-day-old NPK-fertilized tomato plants derived or not from *Trichoderma harzianum* T34-treated seeds, and with or without a previous 72 h application of salt stress (2 l of a 300 mM NaCl solution per 10 plants). Values correspond to relative measurements against the transcripts in tomato leaves from untreated seeds (control) (2^-ΔΔCt^ = 1). Tomato *actin* was used as an internal reference gene. Bars represent the standard deviations of the mean of the three plant pools with three plants each (*n* = 3). Significant effects were determined by a two-way ANOVA (^∗^*P* < 0.05, ^∗∗^*P* < 0.01, ^∗∗∗^*P* < 0.001 for T34, salt and the T34 × salt interaction, ns, no statistical differences). Different letters denote statistical significance for the T34 × salt interaction.

The expression levels of nine genes involved in systemic responses to biotic or abiotic stress were analyzed by qPCR in the aerial part of 35-day-old tomato plants (**Figure 1**). Statistical analysis showed that the combined application of T34 and salt to NPK-fertilized plants caused significant changes in the expression of eight genes out of nine analyzed: *EIN2*, encoding a central component of the ET signaling pathway; *NPR1*, which encodes a key transducer of SA signaling involved in plant defense responses; *AREB2*, encoding a transcription factor (TF) of ABA signaling; *LERBOH1*, involved in ROS production; *APX1*, encoding an ascorbate peroxidase; *SOS1*, which encodes a salt tolerance marker; *ARF1*, encoding a TF that binds to auxin response elements; and *DREB3*, a marker gene involved in tolerance to drought. Compared to NPK-fertilized control, the application of T34 alone increased the expression levels of *NPR1* and *DREB3*, and reduced those of *EIN2, APX1*, and *SOS1*; while the application of salt alone increased the expression of all analyzed genes except *APX1*. A significant T34 effect (pair T34 and T34+salt) as compared to untreated T34 (pair control and salt) plants was detected for all the markers tested with the exception of *APX1* and *DREB3* genes. Similarly, a significant salt effect was observed in the expression of all genes except *APX1, SOS1*, and *ARF1* between salt-treated and untreated plant pairs. The upregulation of *APX1* and the downregulation of *AREB2, NPR1, EIN2, LERBOH1, SOS1, DREB3*, and *ARF1* detected in plants from the T34+salt condition, compared to those treated with salt alone, are compatible with the already noted decaying phenotype observed in T34+salt plants.

**FIGURE 2:**
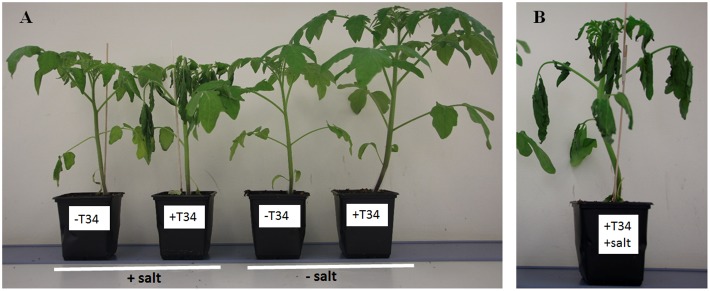
**NPK-fertilized tomato plants.** **(A)** Plants were derived from *T. harzianum* T34-uncoated (-T34) or –coated (+T34) seeds. NPK fertilizer was applied to 24-day-old plants, and salt stress (2 l of a 300 mM NaCl solution per 10 plants) was applied to 32- and 36-day-old plants (+salt) or not (-salt). **(B)** Decayed symptoms in NPK-fertilized tomato plants after the combined application of T34 and salt. The photograph was taken when the plants were 39 days old.

A marked decay was observed when a second salt addition was applied to 36-day-old plants previously treated with T34, NPK and salt (**Figure 2**). The results shown in **Table 2** correspond to 39-day-old plants, and indicate that under NPK fertilization the inoculation of T34 alone led to an increase in all the growth parameters tested with respect to the control plants, with the increases in fresh weight and aboveground dry weight being 35.5 and 47.3%, respectively. However, the inoculation of T34 led to a significant increase in aboveground height when both salt-treated and untreated plants were considered (*P*_T34_). Three days after the second salt application, a significant reduction was detected in height, fresh weight, and aboveground and root dry weights, independently of the presence of T34. Moreover, the combined application of salt and T34 had deleterious effects on the tomato plants (**Figure 2**), with a significant reduction in fresh weight and aboveground and root dry weights. In fact, these last three parameters were reduced by 22.3, 15.9, and 29.4%, respectively, in comparison with those from plants treated with salt alone.

**Table 2:** Growth parameters of NPK-fertilized tomato plants.

Treatment	Height	Diameter	Fresh weight	Aboveground dry weight	Root dry weight
Control	24.59	5.91	14.55 b	1.88 b	0.25 b
T34	26.78	6.60	19.72 c	2.77 c	0.35 c
Salt	21.35	5.59	13.22 a	1.38 a	0.17 a
T34+Salt	20.95	5.67	10.27 a	1.16 a	0.12 a
*P*_T34 × Salt_	ns	ns	^∗∗^	^∗∗^	^∗^
*P*_T34_	^∗^	ns	ns	ns	Ns
*P*_Salt_	^∗∗^	ns	^∗∗∗^	^∗∗∗^	^∗∗∗^


*Data were recorded in plants derived or not from *T. harzianum* T34-treated seeds, and with or without two applications of salt stress (2 l of a 300 mM NaCl solution per 10 plants). NPK fertilizer was applied to 24-day-old plants, and salt was applied to 32- and 36-day old plants. All measurements were taken on 39-day-old plants. Height (cm), diameter (mm), fresh weight (g), canopy dry weight (g), and root dry weight (g). Each value is the mean of 10 biological replicates per condition (*n* = 10). Significant effects were determined by a two-way ANOVA (^∗^*P* < 0.05, ^∗∗^*P* < 0.01, ^∗∗∗^*P* < 0.001) for T34, salt and the T34 × salt interaction, ns, no statistical differences. Different letters denote statistical significance for the T34 × salt interaction.*

### Effect of T34 and Salt Application on Non-NPK-Fertilized Tomato Plants

The leaf photosynthetic rate recorded in unfertilized tomato plants at saturating irradiance was not significantly modified by the treatment with *T. harzianum* T34 relative to the T34-untreated plants (**Table 3**). However, this parameter tended to increase by 28% when plants were inoculated with T34 (*P* = 0.076). Higher stomatal conductance was observed in plants inoculated with T34 compared to the control plants under both non- and salt stress conditions after 3 days (**Table 3**). Salt stress did not decrease stomatal conductance in the salt control plants, although it led to an increase in stomatal conductance in plants inoculated with T34. Furthermore, intercellular CO_2_ concentration in tomato plants decreased with the inoculation of T34.

**Table 3:** Gas-exchange measurements in non-NPK-fertilized tomato plants.

Treatment	A_n_	g_s_	E	C_i_
Control	3.78	44.8 ab	0.66	265
T34	5.45	48.6 b	0.73	251
Salt	4.60	35.7 a	0.45	228
T34+Salt	5.27	64.2 c	0.76	207
*P*_T34 × Salt_	ns	^∗∗^	ns	ns
*P*_T34_	ns	^∗∗∗^	ns	^∗^
*P*_Salt_	ns	ns	ns	ns


*Rate of photosynthesis (A_*n*_), stomatal conductance (g_*s*_), transpiration (E) and intercellular CO_*2*_ concentration (C_*i*_) were recorded in plants derived or not from *T. harzianum* T34-treated seeds, and with or without salt stress (2 l of a 300 mM NaCl solution per 10 plants). Salt was applied to 32-day-old plants. Measurements were taken 72 h after salt application. Each value is the mean of six replicates (*n* = 6), and are expressed in μmol m^-*2*^ s^-*1*^, A_*n*_; mmol m^-*2*^ s^-*1*^, g_*s*_; mmol m^-*2*^ s^-*1*^, E; and μmol mol^-*1*^, Ci. Significant effects were determined by a two-way ANOVA (^∗^*P* < 0.05, ^∗∗^*P* < 0.01, ^∗∗∗^*P* < 0.001) for T34, salt and the T34 × salt interaction, ns, no statistical differences. Different letters denote statistical significance for the T34 × salt interaction.*

**FIGURE 3:**
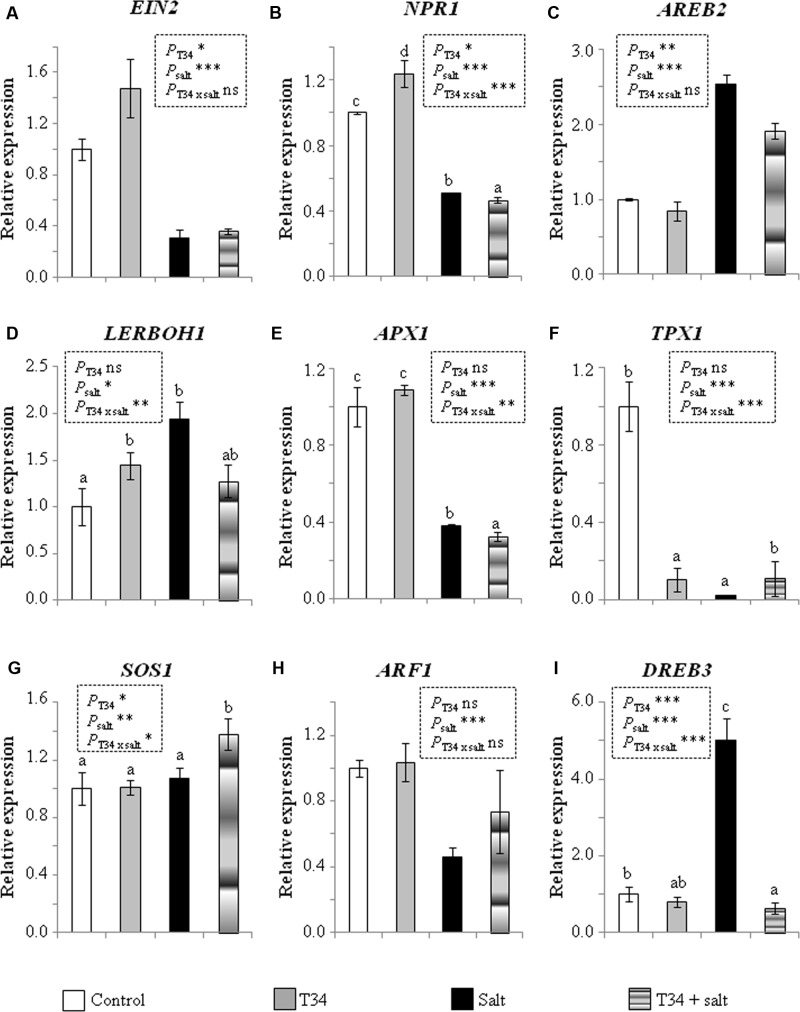
**Relative expression of the genes *EIN*2** **(A)**, *NPR1* **(B)**, *AREB2* **(C)**, *LERBOH1* **(D)**, *APX1* **(E)**, *TPX1* **(F)**, *SOS1* **(G)**, *ARF1* **(H)**, and *DREB3* **(I)** in leaves from 35-day-old non-NPK-fertilized tomato plants derived or not from *T. harzianum* T34-treated seeds, and with or without a previous 72 h application of salt stress (2 l of a 300 mM NaCl solution per 10 plants). The values correspond to relative measurements against the transcripts in tomato leaves from untreated plants (control) (2^-ΔΔCt^ = 1). Tomato *actin* was used as an internal reference gene. Bars represent the standard deviations of the mean of the three plant pools with three plants each (*n* = 3). Bars represent the standard deviations of the mean of the three plant pools with three plants each. Significant effects were determined by a two-way ANOVA (^∗^*P* < 0.05, *P* < 0.01^∗∗^, ^∗∗∗^*P* < 0.001 for T34, salt and the T34 × salt interaction, ns, no statistical differences). Different letters denote statistical significance for the T34 × salt interaction.

**Figure 3** shows the marker gene expression levels detected 72 h after salt application in non-NPK fertilized plants. Unfertilized plants from the T34 and salt treatment did not record the decaying phenotype observed in those NPK-fertilized. Compared to control plants, the application of T34 alone caused expression changes in five out of nine of the genes analyzed, with upregulation of *EIN2, NPR1* and *LERBOH1*, and downregulation of *AREB2* and *TPX1*; while the application of salt alone caused upregulation of *AREB2, LERBOH1* and *DREB3*, and downregulation of the other genes with the exception of *SOS1*. Compared to T34-untreated plants, T34 led to significant changes in the expression of five genes, with an upregulation of *EIN2, NPR1* and *SOS1*, and a downregulation of *AREB2* and *DREB3*. Compared to salt-untreated plants, salt application significantly modified the expression of the nine genes tested, with upregulation of those involved in ROS generation (*LERBOH1*), ABA signaling (*AREB2*), salt and drought tolerance (*SOS1* and *DREB3*), and the downregulation of the ROS scavenging gene *APX1* and the defense- and growth-related genes *EIN2, NPR1, TPX1*, and *ARF1*. Interestingly, the NPK-fertilized plants’ response to salt involved the upregulation of *EIN2, NPR1* and *TPX1*. The combined application of T34 and salt led to a significant downregulation of *NPR1* and *APX1*, accompanied by the significant upregulation of *SOS1* in non-NPK-fertilized tomato plants.

**FIGURE 4:**
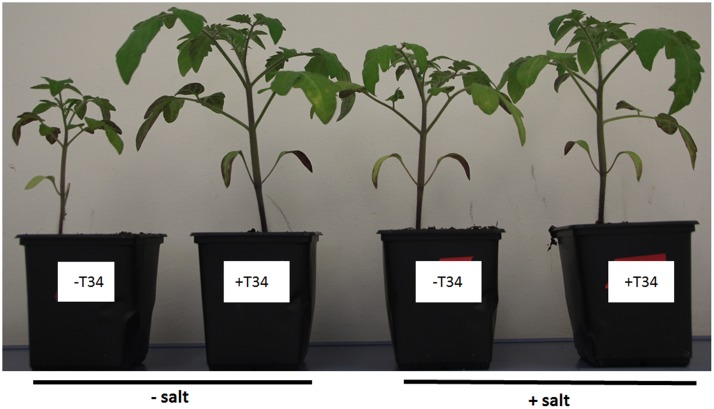
**Non-NPK-fertilized tomato plants.** Plants were derived from *T. harzianum* T34-uncoated (-T34) or -coated (+T34) seeds, and were treated with salt stress (2 l of a 300 mM NaCl solution per 10 plants) at 32 and 36 days (+salt) or not (-salt). The photograph was taken when the plants were 39 days old.

A second application of salt to 36-day-old plants did not have any observable negative symptoms for any of the treatments (**Figure 4**). The growth parameters recorded in these plants 3 days later are shown in **Table 4**. Compared to control plants, height, diameter, fresh weight, and aboveground and root dry weights were increased by *Trichoderma* application; and salt application caused an increase in height (1.8%), fresh weight (5.3%), and root dry weight (34%). T34 significantly increased the five growth parameters analyzed in non-NPK-fertilized plants, while salt reduced all of them, with the exception of root dry weight. Statistical analysis of all plant growth data showed that T34 increased the five growth parameters analyzed in non-NPK-fertilized plants when T34-treated and untreated plants were compared. In addition, the comparison between salt-treated and untreated plants showed that the salt application led to a reduction of all growth parameters, with the exception of root dry. Significant changes were detected for fresh weight and aboveground and root dry weights in the double T34 and salt interaction.

**Table 4:** Growth parameters of non-NPK-fertilized tomato plants.

Treatment	Height	Diameter	Fresh weight	Aboveground dry weight	Root dry weight
Control	12.34	4.24	3.75 a	0.62 ab	0.15 a
T34	15.41	4.65	5.07 b	0.80 b	0.20 ab
Salt	12.56	4.06	3.96 a	0.58 a	0.23 ab
T34+Salt	14.69	4.33	4.87 ab	0.72 ab	0.27 b
*P*_T34 × Salt_	ns	ns	^∗∗^	^∗∗^	^∗^
*P*_T34_	^∗∗^	^∗^	^∗^	^∗^	^∗∗^
*P*_Salt_	^∗∗∗^	^∗∗^	^∗∗∗^	^∗∗∗^	^∗^


*Data were recorded in plants derived or not from *T. harzianum* T34-treated seeds, and with or without two applications of salt stress (2 l of a 300 mM NaCl solution per 10 plants). Salt was applied to 32- and 36-day old plants. All measurements were taken on 39-day-old plants. Height (cm), diameter (mm), fresh weight (g), canopy dry weight (g), and root dry weight (g). Each value is the mean of 10 biological replicates per condition (*n* = 10). Significant effects were determined by a two-way ANOVA (^∗^*P* < 0.05, ^∗∗^*P* < 0.01, ^∗∗∗^*P* < 0.001) for T34, salt and the T34 × salt interaction, ns, no statistical differences. Different letters denote statistical significance for the T34 × salt interaction.*

### Changes in Marker Gene Expression Associated to Salt Stress

**FIGURE 5:**
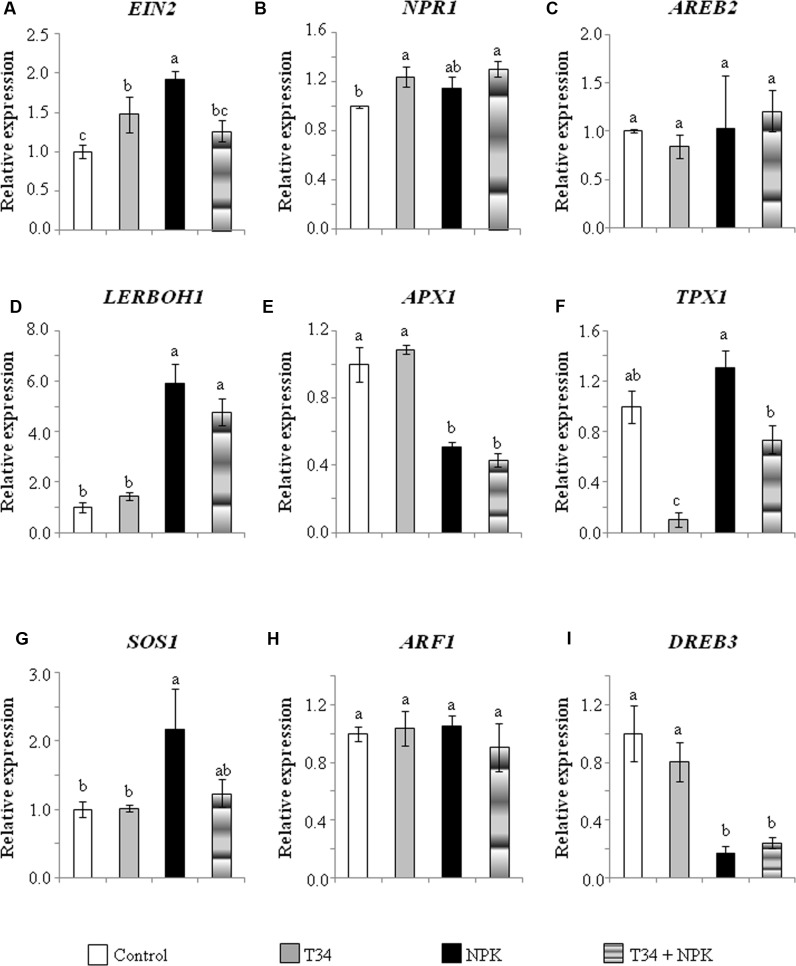
**Relative expression of the genes *EIN*2** **(A)**, *NPR1* **(B)**, *AREB2* **(C)**, *LERBOH1* **(D)**, *APX1* **(E)**, *TPX1* **(F)**, *SOS1* **(G)**, *ARF1* **(H)**, and *DREB3* **(I)** in leaves from 35-day-old salt-untreated tomato plants derived or not from *T. harzianum* T34-treated seeds, and with or without a previous fertilization with NPK (1 | of a solution containing 2 g/l Multi-Feed NPK fertilizer per 10 plants). Values correspond to relative measurements against the transcripts in tomato leaves from untreated seeds (control) (2^-ΔΔCt^ = 1). Tomato *actin* was used as an internal reference gene. Bars represent the standard deviations of the mean of the three plant pools with three plants each (*n* = 3). Significant effects were determined by a one-way ANOVA followed by Tukey’s test. For each gene, different letters represent significant differences (*P* < 0.05).

**FIGURE 6:**
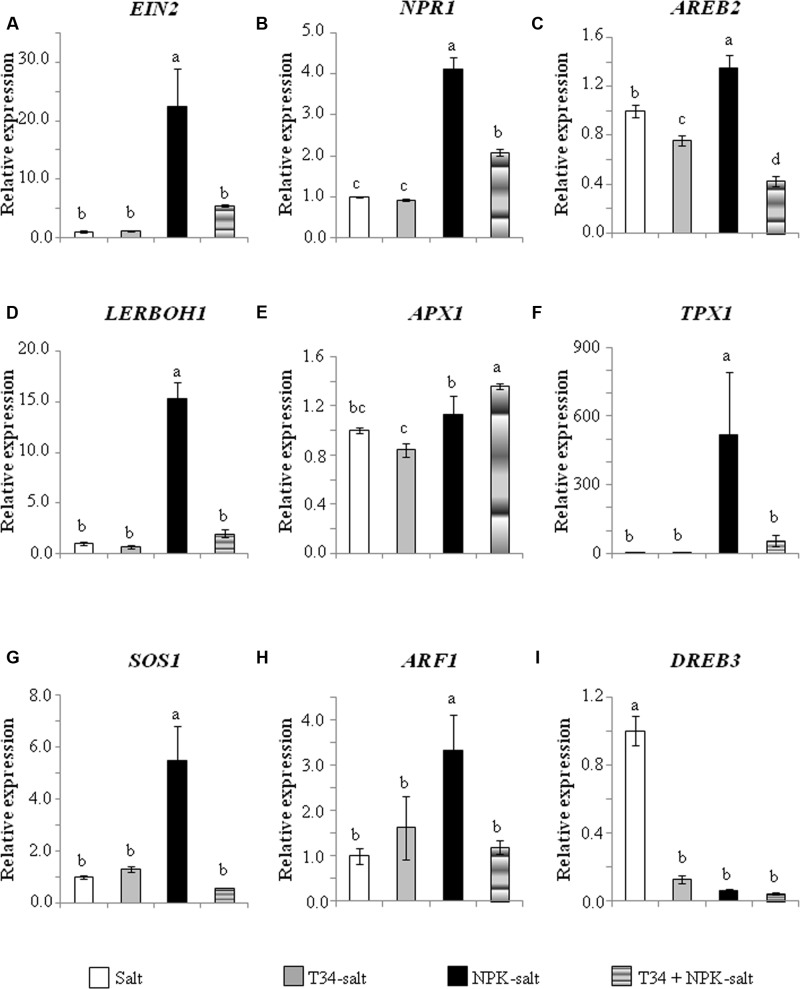
**Relative expression of the genes *EIN*2** **(A)**, *NPR1* **(B)**, *AREB2* **(C)**, *LERBOH1* **(D)**, *APX1* **(E)**, *TPX1* **(F)**, *SOS1* **(G)**, *ARF1* **(H)**, and *DREB3* **(I)** in leaves from 35-day-old salt-treated tomato plants derived or not from *T. harzianum* T34-treated seeds, and with or without a previous fertilization with NPK (1 | of a solution containing 2 g/l Multi-Feed NPK fertilizer per 10 plants). Values correspond to relative measurements against the transcripts in tomato leaves from untreated seeds (Salt) (2^-ΔΔCt^ = 1). Tomato *actin* was used as an internal reference gene. Bars represent the standard deviations of the mean of the three plant pools with three plants each (*n* = 3). Significant effects were determined by a one-way ANOVA followed by Tukey’s test. For each gene, different letters represent significant differences (*P* < 0.05).

The marker gene expression levels obtained from the two *in vivo* experiments were analyzed considering T34 and NPK individual treatments as well as the combination of both in the absence and presence of salt stress. In plants without salt (**Figure 5**), the application of T34 caused a significant upregulation of *EIN2* and *NPR1* but a downregulation of *TPX1* with respect to the control (untreated) condition; while in plants treated with NPK, a significant increase in the expression levels of *EIN2, LERBOH1*, and *SOS1* and a decrease in those of *APX1* and *DREB3* were observed. Also in absence of salt, the combined application of T34 and NPK gave a significant increase of *NPR1* and *LERBOH1* expression levels and a decrease in those of *APX1* and *DREB3*. Under salt stress (**Figure 6**), plants treated with T34 only showed significant downregulation of *AREB2* and *DREB3* and, with the exception of *APX1*, NPK application resulted in significant expression changes in all genes analyzed, seven of them being upregulated and only *DREB3* downregulated. Plants treated with T34+NPK in the presence of salt, showed increased and decreased expression levels of *APX1* and *AREB2*, respectively. This expression profile proved to be significant not only in comparison with the control condition but with that from plants treated with T34 alone and NPK alone.

## Discussion

Plants are often subject to chemical fertilization in order to increase crop productivity in modern agriculture (Mulvaney et al., 2009; Kraiser et al., 2011). It is also recognized that strains from *Trichoderma* spp. are able to colonize the rhizosphere and promote plant growth (Shoresh et al., 2010; Hermosa et al., 2012). Moreover, strains of *Trichoderma* can help plants to overcome adverse environmental conditions (Mastouri et al., 2010; Rubio et al., 2014). Here, we have evaluated the response of tomato plants to *T. harzianum* T34, salt stress, and their combination, under two different chemical fertilization conditions. The fertilizer NPK was applied to 24-day-old plants in a solution at the agronomical recommended dosage of 0.2 g per plant. Thus, as expected, the 39-day-old control plants that were NPK-fertilized recorded higher growth parameters (**Table 2**) than those unfertilized (**Table 4**). These two different phenotypes were consistent with the higher gas-exchange values recorded for NPK-fertilized control plants (**Table 1**) in comparison to those from the unfertilized control (**Table 3**). Although T34 treatment compared to control plants increased most of the growth parameters in both NPK-fertilized and unfertilized plants, only the height was significantly changed in NPK-fertilized plants by the T34 application while the five growth parameters were significantly modified in non-fertilized plants (*P*_T34_). Curiously, T34 caused a significant decrease in intercellular CO_2_ concentration in unfertilized plants in step with higher stomatal conductance and photosynthetic rates (**Table 3**). The marked *Trichoderma* effect observed in unfertilized plants with respect to the NPK-fertilized ones may be because their scope for improvement was wider.

Salinity negatively affects most plant growth phases and alters development and leaf structure (Parida and Das, 2005). Moreover, ion imbalances are significantly less detrimental during vegetative growth (Munns, 2002; Park et al., 2016). In our study, salt stress generally had a negative effect on the growth and development of adult tomato plants at the end of the two experiments (**Tables 2**, **4**). However, after 72 h the effects of salt application to NPK-fertilized plants included reduced stomatal conductance and transpiration, as well as the inhibition of photosynthesis, while in unfertilized plants no significant changes were observed (**Tables 1**, **3**). These different gas-exchange responses could be a physiological disadvantage of NPK application under salt stress, because as previously reported (Craufurd et al., 2008), the capacity plants have to modulate leaf-gas exchange leads to optimal CO_2_ assimilation rates, which are associated with a higher tolerance to abiotic stress (Zandalinas et al., 2016b). A recent study has also shown that changes in the nitrogen metabolism of *Populus simonii* affect its resistance to moderate salt stress, and the application of salt reduces the photosynthetic rate more sharply in *P. simonii* plants fed with ammonium than when fed with nitrate (Meng et al., 2016). Interferences in mycorrhiza-induced resistance against *Botrytis cinerea* in tomato plants due to the availability of nitrogen have also been described recently (Sánchez-Bel et al., 2016). These last authors indicate that plants that have suffered a short period of nitrogen starvation appear to react by reprogramming their metabolic and genetic responses to prioritize abiotic stress tolerance. The fertilizer NPK used in our study contains nitrogen in ammonium and nitrate forms, and the gas-exchange parameters indicate that unfertilized plants are more tolerant to salt stress or are more capable of adapting than the NPK-fertilized ones.

The NPK-fertilized and non-fertilized plants showed a contrasting ability to tolerate salt stress when they were from T34-treated seeds. Firstly, the decaying phenotype of fertilized plants in response to the salt application was not observed in those not fertilized. In the experiment performed with unfertilized plants, for the T34+salt treatment the effect of T34 on gas-exchange parameters such as stomatal conductance and transpiration predominated over that of salt, and the induced stomatal aperture was associated with CO_2_ assimilation, which tended to increase significantly. However, an apparent inconsistency seemed to exist in the NPK-fertilized plants in the T34+salt treatment, as T34 or salt alone reduced gas exchange, but the combination of T34 and salt simultaneously enhanced stomatal conductance and intercellular CO_2_ concentration, and suppressed photosynthesis. It is worthwhile noting that at the end of the experiment, when NPK-fertilized plants were 39 days old, the 10 plants of the T34+salt set were damaged by the salt application (**Figure 2**). Thus, in order to understand this increased sensitivity to salt stress, the molecular responses of plants from the different treatments were analyzed (**Figures 1**, **3**, **5**, **6**).

Several studies have reported the transcriptomic responses of plants to the presence of a root colonizing *Trichoderma* biocontrol strain (Alfano et al., 2007; Morán-Diez et al., 2012; Domínguez et al., 2016), but little is known about tomato plant responses to T34+salt, and the role played by a chemical fertilizer when these two treatments are combined. It has also been reported that after the initial *T. harzianum*-plant interaction process, when root colonization is well-established, this fungus induces minor changes in the plant transcriptome, as plant responses decline over time because the stimulus is less intense (Lorito et al., 2010; Domínguez et al., 2016). The significant expression changes detected for the nine genes involved in ABA-, SA-, ET- or auxin-responsive pathways, production and the scavenging of ROS, and tolerance to salt and dehydration stresses, show that the tomato plant responds not only to *T. harzianum*, but to the T34+salt combination, which is clearly differentiated in NPK-fertilized and unfertilized plants.

The marked upregulation of *AREB2, LERBOH1*, and *DREB3* and the downregulation of *APX1* observed independently of the chemical fertilizer application (**Figures 1**, **3**) in plants treated only with salt is consistent with a salt signaling response (Mittler and Blumwald, 2015; Park et al., 2016). ABA is particularly important for abiotic stress recognition, as guard cell movement is regulated by this hormone, and the stomatal closure generates a CO_2_ deficit and reduces photosynthesis, leading to increased ROS production (Zhu, 2002; Park et al., 2016). In addition, ABA induces the synthesis of dehydration tolerance proteins with general metabolic adjustments (Park et al., 2016), which is consistent with the *DREB3* upregulation observed. However, in salt-stressed plants from the treatment T34+NPK, the expression levels of *AREB2* and *APX1* were respectively much lower and higher than those that could be expected for plants responding to a salt stress (**Figure 6**).

The different regulation of *EIN2, NPR1, TPX1, SOS1*, and *ARF1* observed in response to salt between NPK-fertilized and unfertilized plants shows they have a different sensitivity to salt stress. The SOS pathway plays an important role in regulating Na^+^/K^+^ homeostasis and salt tolerance, and includes SOS1, a plasma membrane-localized Na^+^/H^+^ antiporter that mediates Na^+^ efflux from the roots, and the loading of Na^+^ ions in the xylem (Shi et al., 2000; Munns, 2002). *SOS1* is unstable under normal growth conditions, but its stabilization is mediated by salt, dehydration and ROS (Shi et al., 2002; Chung et al., 2008). Considering that unfertilized plants did not record lower photosynthetic rates and growth parameters, and the upregulation of *SOS1* did not occur in response to salt application compared to control plants, it may be assumed that the salt concentration used was not particularly stressful, or that salt adaptation changes may already have occurred during the 72 h elapsed since salt application. The opposite response in NPK-fertilized plants to the same salt concentration suggests that this fertilizer increases the sensitivity of tomato plants to salt stress. Moreover, the low expression level of *SOS1*, although no significantly with respect to that of its salt application control, is in tune with that of *AREB2* detected in salt-stressed plants from the T34+NPK treatment (**Figure 6**). ET signaling regulates plant growth and development, and EIN2, a key protein in the ET signaling pathway, is required for salt tolerance (Gazzarrini and McCourt, 2001; Lei et al., 2011). Although mutations leading to reduced ET emission also increase tolerance to abiotic stresses due to the upregulation of stress responsive ABA-dependent genes (Dong et al., 2011), the *EIN2* upregulation observed in response to salt stress in the NPK-fertilized plants was accompanied by the upregulation of *SOS1* and *DREB3*, two ABA-dependent genes. Moreover, it is well-known that a lack of EIN2 function leads to extreme salt sensitivity, while an overexpression of its C-terminus suppresses it (Alonso et al., 2003; Lei et al., 2011). EIN2 is a point of crosstalk for several different pathways, and melon plants have shown that *EIN2* expression is influenced by auxin and ABA (Gao et al., 2013). In both experiments, our results show that a correlation between expression levels of *EIN2* and *ARF1* always occurred, but this correlation was not always observed between *EIN2* and *AREB2*. It has been reported that salt alters the expression of auxin-responsive genes (Liu et al., 2013; Park et al., 2016), while auxin overproduction in *Medicago truncatula* increases its tolerance to high saline stress (Bianco and Defez, 2009). This fact could serve to explain the absence of significant expression changes in *ARF1* in salt-unstressed plants (**Figure 5**).

As a key regulator of SAR, NPR1 is essential for SA signaling transduction (Cao et al., 1994), and it has recently been reported that this TF is pivotal for controlling Na^+^ entry into the root and its transport into the shoot, as well as for preventing potassium loss, with NPR1-dependent SA signaling being crucial for salt and oxidative stress tolerance in *Arabidopsis* (Jayakannan et al., 2015). Thus, the *NPR1* upregulation observed in NPK-fertilized plants in response to salt is to be expected, as these plants recorded reduced growth, but tolerance to the salt stress applied. In these plants, *TPX1* was coordinately upregulated with *NPR1*, as would be expected for a gene encoding a cell-wall peroxidase activated by SA signaling (Lucena et al., 2003).

It has recently been reported that ABA is required for the accumulation of APX1 during a combination of water deficit and heat stress (Zandalinas et al., 2016a). This study has also suggested that mutants impaired in ABA signaling recorded reduced growth and biomass, but better acclimation to this combined stress due to stomatal closure accompanied by higher levels of H_2_O_2_ and reduced accumulation of the ROS scavenger APX1. In our study, NPK-fertilized plants from the treatment T34+salt were unable to acclimate to the salt stress applied (**Figure 2**). The increased intercellular CO_2_ and slight increases in stomatal conductance and transpiration compared to salt-treated plants could be explained by the reduced expression of *AREB2* and *LERBOH1*, and the increase detected for *APX1.* These results are consistent with the significant transcription changes observed for *AREB2* and *APX1* in T34+NPK salt-stressed plants compared with those detected in T34+salt and NPK+salt individual treatments and the corresponding salt alone control (**Figure 6**). The detected downregulation of the salt tolerance marker *SOS1* is consistent with the decaying phenotype observed in the NPK-fertilized plants from the T34+salt treatment, which recorded a significantly reduced biomass at the end of the experiment, when the two salt applications were performed.

In order to understand the processes that triggered the high sensitivity to salt among NPK-fertilized plants from the T34+salt combination, an identical experiment was performed in the absence of a chemical fertilizer. Although the unfertilized plants from the T34+salt treatment did not record significant expression changes for the ET and ABA markers, these plants had reduced SA signaling and ROS production, and showed a tolerance to salt, which was marked by a significant upregulation of *SOS1*. As indicated above, the strong downregulation of *APX1* might serve to maintain high enough levels of ROS to trigger the salt tolerance response. These results are consistent with the highest stomatal aperture, even higher than that detected in the T34 treatment, and the higher aboveground and root weights detected at the end of the experiment. An increase in biomass was previously reported for unfertilized tomato plants grown from *T. parareesei* T6-treated seeds under salt stress (Rubio et al., 2014).

In sum, NPK-fertilized tomato plants are more sensitive to salt stress than their unfertilized counterparts. The application of *T. harzianum* T34 increased growth and the tolerance to salt of unfertilized plants. However, the overstimulation of growth and development triggered by the fertilizer NPK in T34-treated plants led to a deregulation of the phytohormone networking, and the plants were unable to adapt to an environmentally adverse scenario. In fact, these plants did not record a significantly reduced height or stem diameter, but instead a decaying phenotype with a significantly low fresh weight and aboveground and root dry weights.

## Author Contributions

WB and RH conceived, designed and performed the greenhouse experiments; MR, RV, and FG-A performed the laboratory experiments; WB, RV, MR, RH, and EM analyzed the data; RM and EM contributed reagents/materials/analysis tools; RH, EM, and WB wrote the paper.

## Conflict of Interest Statement

The authors declare that the research was conducted in the absence of any commercial or financial relationships that could be construed as a potential conflict of interest.
