# Supplementary material for: The Combination of Trichoderma harzianum and Chemical Fertilization Leads to the Deregulation of Phytohormone Networking, Preventing the Adaptive Responses of Tomato Plants to Salt Stress
Source: Front Plant Sci. 2017 Mar 2;8:294. doi: 10.3389/fpls.2017.00294 (PMC5332374; doi:10.3389/fpls.2017.00294)
Supplement: Supplementary file 1 [file Table_1.DOCX]

**TABLE S1. Primers used for quantitative Real-time PCR (qPCR) analysis.**

| **Name** | **Sequences (5’ → 3’)** | **Encoding (gene)** |
| --- | --- | --- |
| EIN2-fw | GTTGCTAAGTGATGCTGTA | Ethylene-insensitive protein 2 *(EIN2)* |
| EIN2-rev | GTTGCTAAGTGATGCTGTA |  |
| NPR1-fw | GGCGGACAACCTGCGTCAAC | Nonexpressor of pathogenesis-related gene *(NPR1)* |
| NPR1-rev | GCTCTCGTGGTCTGGCAAGC |  |
| AREB2-fw | GCTCAACAGGAGGAGTGG | ABA-responsive element binding protein 2 (*AREB2*) |
| AREB2-rev | CATCAACAGTCTTATGACTCAG |  |
| LeRBOH1-fw | GTCAGGCTTCTACAGAAAAC | NADPH oxidase (*LeRBOH1*) |
| LeRBOH1-rev | GTTGATTACAGTAGCCGGTTC |  |
| APX1-fw | GGTCTTGACATTGCTCTCA | Ascorbate peroxidase (*APX1*) |
| APX1-rev | CTGGTGGCTCTGGCTTGTCC |  |
| TPX1-fw | GCTTTGTCAGGGGTTGTGAT | Cell-wall peroxidase (*TPX1*) |
| TPX1-rev | TGCATCTCTAGCAACCAACG |  |
| SOS1-rev | GGTGGACTTCTAAGCGCTAC | Salt overlay sensibility 1 (*SOS1*) |
| SOS1-fw | GAAATTTGATGACAGCTCCCC |  |
| SlARF1-fw | GCAGCAACACCTACAAC | Auxin responsive factor *(ARF1)* |
| SlARF1-rev | ACAGGAGACTTCCACATTC |  |
| DREB3-fw | GAATCAGTTAACCCCAATTCA | Dehydration-responsive element-binding protein 3 (*DREB3*) |
| DREB3-rev | GCTTCGTGGGTTTTGGTGGCG |  |
| Actin-fw | CACCACTGCTGAACGGGAA | Actin (*ACT*) |
| Actin-rev | GGAGCTGCTCCTGGCACTTT |  |

For each primer pair, efficiency was next to 100%.
